# Cell cycle shift from G0/G1 to S and G2/M phases is responsible for increased adhesion of calcium oxalate crystals on repairing renal tubular cells at injured site

**DOI:** 10.1038/s41420-018-0123-9

**Published:** 2018-11-21

**Authors:** Supaporn Khamchun, Visith Thongboonkerd

**Affiliations:** 1 Medical Proteomics Unit, Office for Research and Development, Faculty of Medicine Siriraj Hospital, Mahidol University, Bangkok, Thailand; 2 Department of Immunology and Immunology Graduate Program, Faculty of Medicine Siriraj Hospital, Mahidol University, Bangkok, Thailand; 3 Center for Research in Complex Systems Science, Mahidol University, Bangkok, Thailand

## Abstract

Renal tubular cell injury can enhance calcium oxalate monohydrate (COM) crystal adhesion at the injured site and thus may increase the stone risk. Nevertheless, underlying mechanism of such enhancement remained unclear. In the present study, confluent MDCK renal tubular cell monolayers were scratched to allow cells to proliferate and repair the injured site. At 12-h post-scratch, the repairing cells had significant increases in crystal adhesion capacity and cell proliferation as compared to the control. Cell cycle analysis using flow cytometry demonstrated that the repairing cells underwent cell cycle shift from G0/G1 to S and G2/M phases. Cyclosporin A (CsA) and hydroxyurea (HU) at sub-toxic doses caused cell cycle shift mimicking that observed in the repairing cells. Crystal-cell adhesion assay confirmed the increased crystal adhesion capacity of the CsA-treated and HU-treated cells similar to that of the repairing cells. These findings provide evidence indicating that cell cycle shift from G0/G1 to S and G2/M phases is responsible, at least in part, for the increased adhesion of COM crystals on repairing renal tubular cells at the injured site.

## Introduction

Development of kidney stone disease requires intense binding of causative crystalline particles to renal tubular epithelium, leading to retention and invasion of these crystals into renal interstitium^1–3^. The most common causative crystal type found in 70–80% of stone formers (patients with kidney stone(s)) is calcium oxalate monohydrate (COM)^4^. Under normal physiologic state, most of these crystals formed inside renal tubular lumens can be eliminated through renal tubular fluid flow and expelled into the urine^5,6^. The rest of them can be endocytosed into renal tubular cells and degraded via endolysosomes^7,8^. Several lines of recent evidence from both in vitro and in vivo studies have shown that renal tubular cell injury can enhance crystal binding at the injured site and thus may increase the stone risk^9–13^. Nevertheless, mechanisms underlying such enhancement remained unclear.

Because renal tubular epithelial cells can repair the injured epithelial line by cell proliferation, we thus hypothesized that cell proliferation and cell cycle modulation during tissue repair process may be involved in the increased crystal adhesion capacity at the injured locale. Our hypothesis was then addressed by various functional investigations, i.e., microscopic examination, scratch assay, crystal-cell adhesion assay, cell death and proliferation assay, immunofluorescence staining, propidium iodide staining, flow cytometry, and cell cycle analysis. Finally, the obtained data were validated by using cyclosporin A (CsA) and hydroxyurea (HU), which are the cell cycle modifiers that could mimic cell proliferation and cell cycle shift that were found in initial experiments (from G0/G1 into S and G2/M phases for CsA^14–16^ and from G0/G1 into S phase for HU^17–19^).

## Results

### Enhanced crystal-cell adhesion in the repairing cell monolayers

**Fig. 1:**
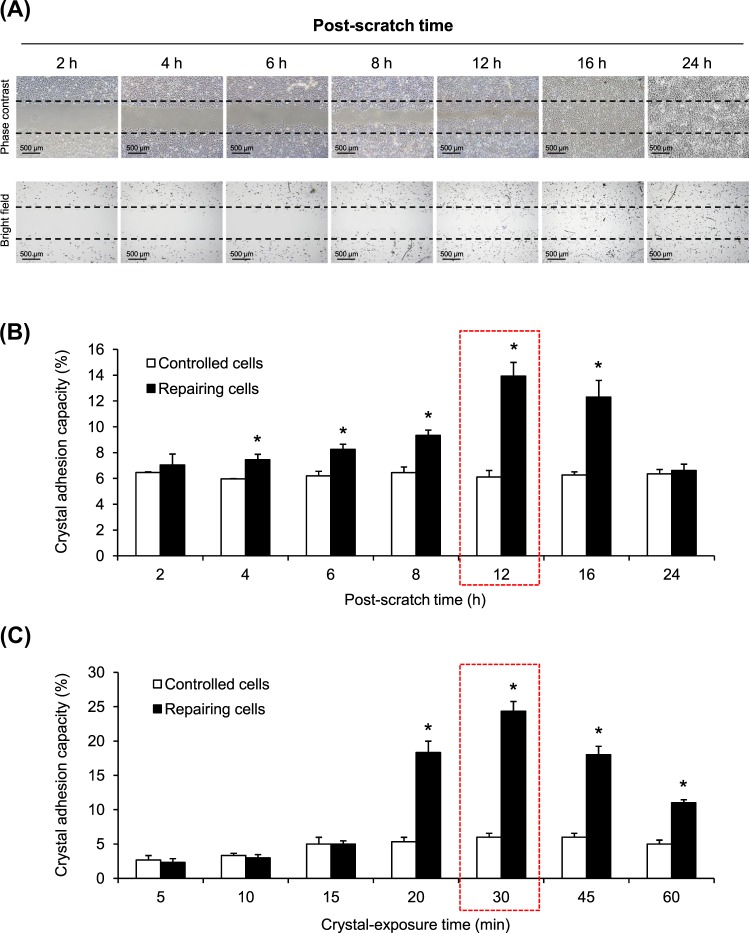
Optimization of crystal-cell adhesion assay to evaluate repairing cells. **a** Multiple mesh-like scratches were made on MDCK confluent monolayer to generate repairing cells, whereas the non-scratched monolayer served as the control. At 2-, 4-, 6-, 8-, 12-, 16-, and 24-h post-scratch, crystal adhesion assay was performed with a fixed crystal-exposure time at 60 min following the standard protocol. Micrographs were taken by using a phase contrast microscope (original magnification = ×40 in all panels). **b** Crystal adhesion capacity of the cells was examined from at least 15 randomized high-power fields (HPFs) in each well. **c** Crystal-cell adhesion assay was performed at a fixed post-scratch time-point (12 h), whereas crystal-exposure time was varied at 5, 10, 15, 20, 30, 45, and 60 min. Each bar represents mean ± SEM of the data obtained from three independent experiments. *p < 0.05 vs. control

Initially, the optimal post-scratch time-point for crystal-cell adhesion assay was defined for this present study addressing effects of tissue repair on crystal adhesion at the injured site. The data showed that crystal adhesion capacity of the repairing cells was significantly increased in the repairing cell monolayers at almost all post-scratch time-points as compared to the controlled cell monolayers (Fig. 1a, b). In the repairing cell monolayers, such increase was progressive from 2- to 12-h post-scratch (maximal at 12 h). Thereafter, such enhancement was diminished at 16-h post-scratch and the crystal adhesion capacity of the repairing cell monolayers returned to the basal level at 24-h post-scratch, when tissue repair was complete (Fig. 1a, b). Next, we defined the optimal crystal-exposure time for this assay. The data showed that exposing the cell monolayers to the crystals for 30 min offered maximal degree of the increase of crystal adhesion capacity of the injured cells (Fig. 1c). Therefore, the post-scratch time-point at 12 h and crystal-exposure time of 30 min were used as the optimal conditions for all subsequent experiments.

### Increased cell proliferation in the repairing cell monolayers

**Fig. 2:**
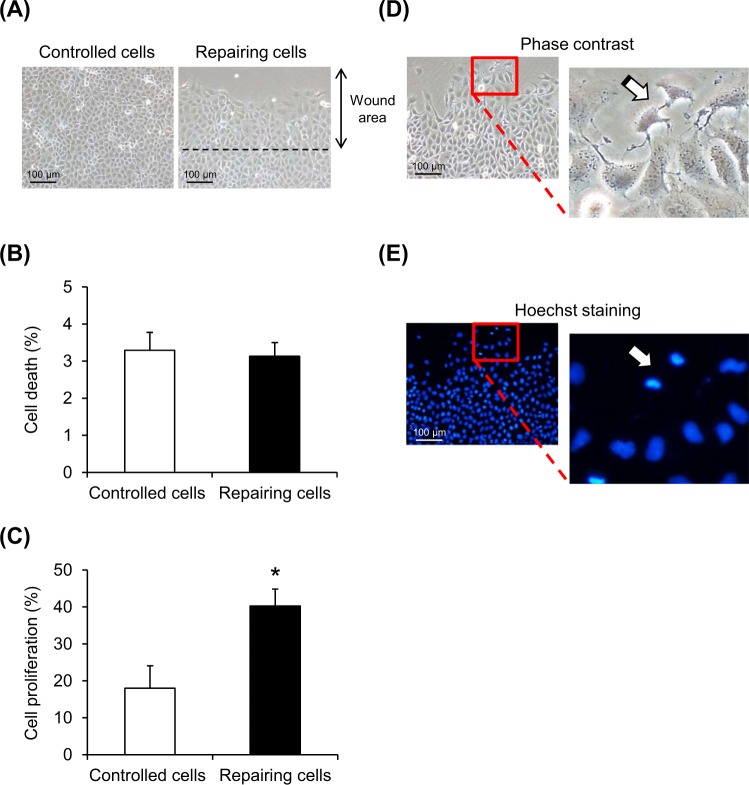
Enhanced cell proliferation in repairing cells. At optimal post-scratch time-point (12 h) and crystal-exposure time (30 min), the repairing and controlled cells were subjected to morphological examination (**a**), cell death assay (**b**), and cell proliferation assay (**c**). Phase contrast microscopy (**d**) and immunofluorescence staining of cellular nuclei (**e**) were also performed to demonstrate dividing cells. Original magnification =×200 for all panels. Each bar represents mean ± SEM of the data obtained from three independent experiments. *p < 0.05 vs. control

Using the optimal post-scratch time-point and crystal-exposure time as defined above, cell death and proliferation were analyzed (Fig. 2a). Quantitative data showed that while cell death was comparable between the controlled and repairing cell monolayers (Fig. 2b), cell proliferation was markedly increased in the repairing cell monolayers (Fig. 2c). Cell proliferation was then highlighted by zoom-in imaging of phase contrast and fluorescence micrographs using Hoechst dye to stain nuclei of the cells (Fig. 2d, e).

### Cell cycle shift in the repairing cell monolayers

**Fig. 3:**
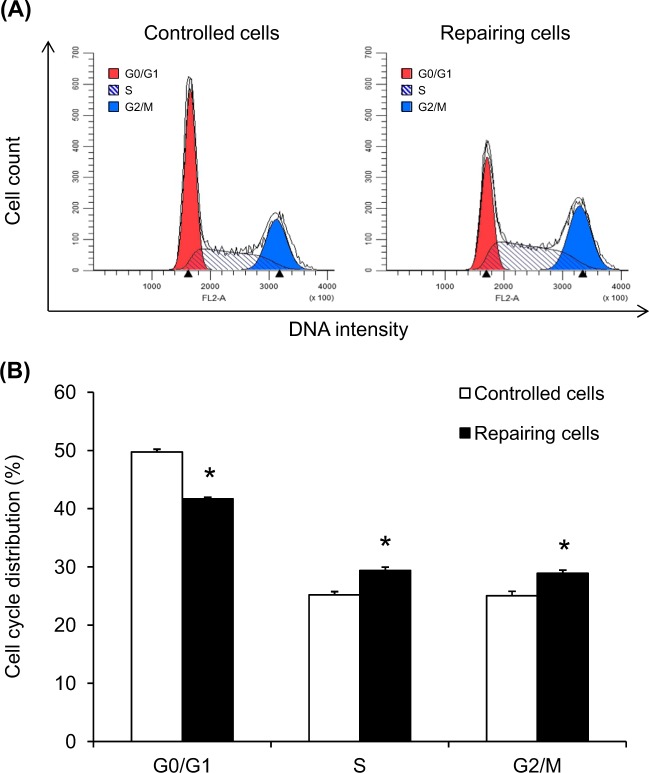
Cell cycle shift in repairing cells. At optimal post-scratch time-point (12  h) and crystal-exposure time (30  min), the repairing and controlled cells were subjected to cell cycle analysis using flow cytometry. **a** Representative histogram of the gated cells in the G0/G1, S, and G2/M phases. **b** Quantitative analysis of distribution or proportion of the cells in each phase was performed from at least 10,000 cells per sample. Each bar represents mean ± SEM of the data obtained from three independent experiments. *p < 0.05 vs. control

The distribution of cell cycle phases in repairing cells at 12-h post-scratch was analyzed comparing to the controlled cells using flow cytometry (Fig. 3a). Quantitative data revealed that repairing cells had reduced distribution in the G0/G1 phase but increased their distribution in both S and G2/M phases (Fig. 3b), indicating that the repairing cells underwent cell cycle shift from G0/G1 to S and G2/M phases.

### Cell cycle shift by CsA mimicked the effect found in the repairing cell monolayers

**Fig. 4:**
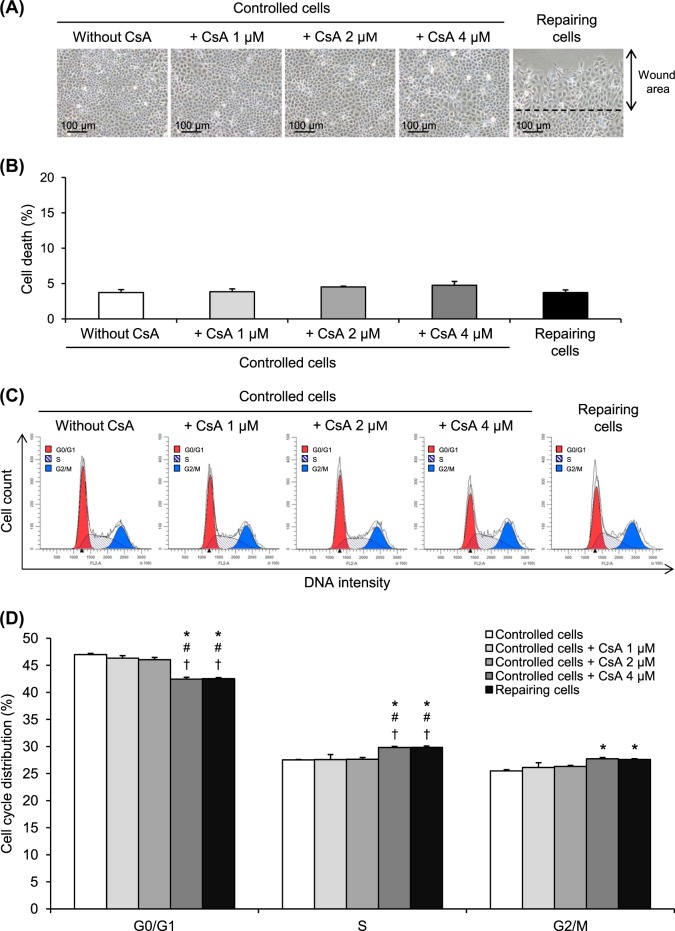
Cell cycle shift by CsA at non-toxic doses. **a** The confluent cell monolayers were treated with 1, 2, or 4 µM CsA for 12 h. Original magnification = ×200 for all panels. **b** The cells were then subjected to cell death analysis comparing to repairing and controlled cells. **c**,**d** Cell cycle analysis was performed on CsA-treated cells compared to repairing and controlled cells. Each bar represents mean ± SEM of the data obtained from three independent experiments. *p < 0.05 vs. controlled cells; ^#^*p* < 0.05 vs. 1 µM CsA-treated cells; ^†^*p* < 0.05 vs. 2 µM CsA-treated cells

CsA, which is a cell cycle modifier^14–16^, was employed for validation of our initial results. Because this compound has potential cytotoxic effects, we thus used the non-toxic doses and then confirmed that the dose range used (1–4 µM) was not toxic to the cells. Microscopic examination showed that the cell morphology looked normal after treating the cells with 1–4 µM CsA for 12 h (Fig. 4a). Additionally, cell death assay revealed no significant increase in cell death after treatment with 1–4 µM CsA comparing to the controlled and repairing cells (Fig. 4b). Cell cycle analysis using flow cytometry demonstrated that CsA at 4 µM caused significant cell cycle shift from G0/G1 to S and G2/M phases similar to the cell cycle shift observed in the repairing cells (Fig. 4c, d). Therefore, CsA at 4 µM was then used for further validation.

### Enhanced crystal-cell adhesion by CsA

**Fig. 5:**
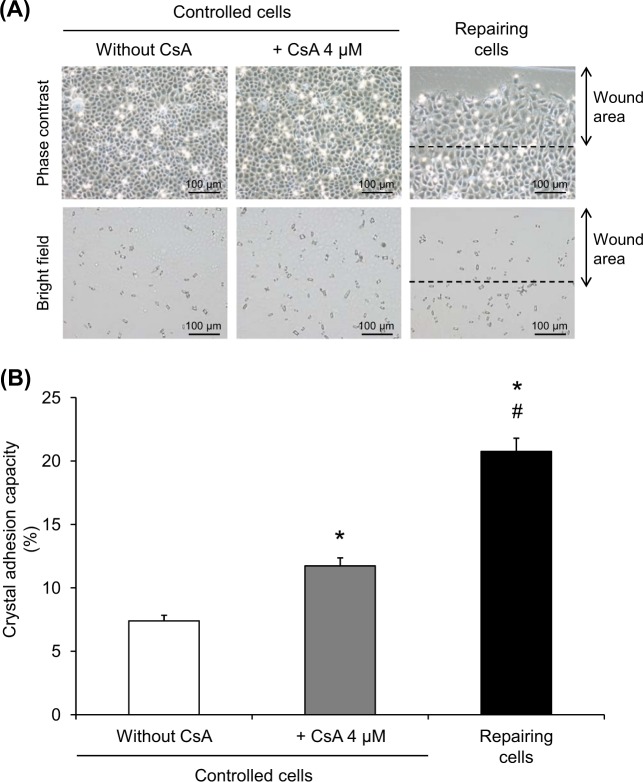
Enhanced crystal adhesion capacity of CsA-treated cells. **a** COM crystal-cell adhesion assay was performed on the controlled, repairing, and CsA-treated cells at 12-h post-scratch or post-treatment. Micrographs were taken by using a phase contrast microscope (original magnification  = ×200 in all panels). **b** Crystal adhesion capacity of the cells was examined from at least 15 randomized high-power fields (HPFs) in each well. Each bar represents mean ± SEM of the data obtained from three independent experiments. *p < 0.05 vs. control; ^#^*p* < 0.05 vs. 4 µM CsA-treated cells

To finally confirm that cell cycle shift from G0/G1 to S and G2/M phases could enhance crystal adhesion capacity, the cell monolayers were treated with 4 µM CsA for 12 h and crystal-cell adhesion assay was performed. The remaining crystals adhered onto the cells after crystal exposure for 30 min were counted from micrographs taken (Fig. 5a) and quantitative data are shown in Fig. 5b. The results revealed that CsA treatment significantly enhanced crystal adhesion capacity of renal tubular cells, although with less degree as compared to the effect observed in the repairing cells (Fig. 5).

### Enrichment of S phase by HU mimicked the effect found in the repairing cell monolayers

**Fig. 6:**
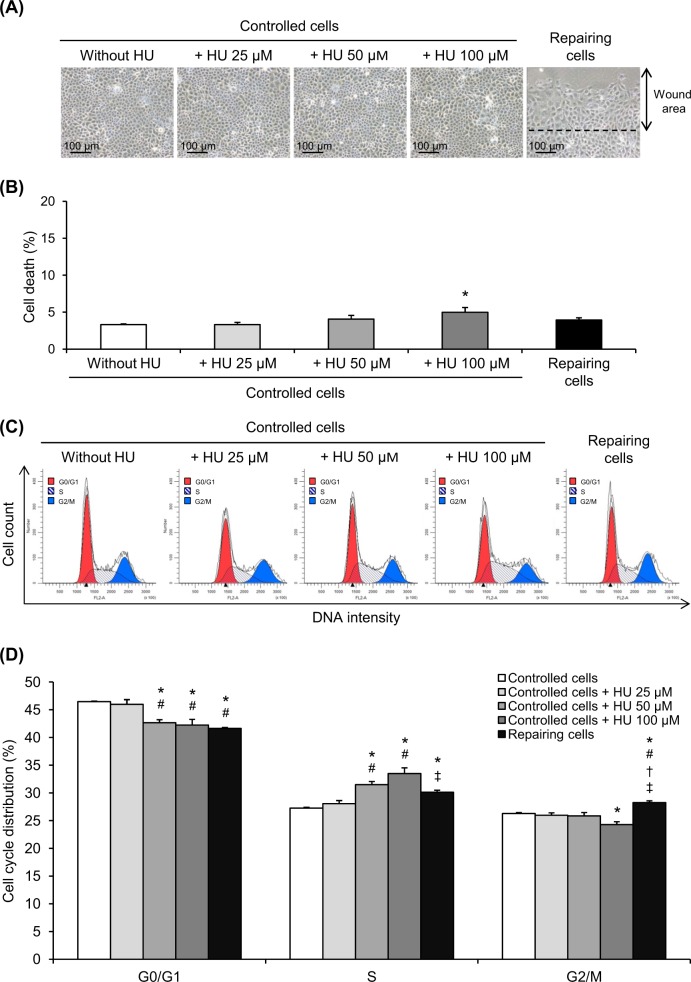
Enrichment of S phase by HU. **a** The confluent cell monolayers were treated with 25, 50, or 100 µM HU for 12 h. Original magnification = ×200 for all panels. **b** The cells were then subjected to cell death analysis comparing to repairing and controlled cells. **c**, **d** Cell cycle analysis was performed on HU-treated cells compared to repairing and controlled cells. Each bar represents mean ± SEM of the data obtained from three independent experiments. *p < 0.05 vs. controlled cells; ^#^*p* < 0.05 vs. 25 µM HU-treated cells; ^†^*p* < 0.05 vs. 50 µM HU-treated cells; ^‡^*p* < 0.05 vs. 100 µM HU-treated cells

HU, which is another cell cycle modifier^17–19^, was also used for validation. Cell morphology (Fig. 6a) and cell death assay (Fig. 6b) showed that HU at 25–50 µM did not cause morphological change and significant increase in cell death. However, HU at a higher dose (100 µM) caused a slight increase in cell death (Fig. 6b). Cell cycle analysis using flow cytometry demonstrated that HU at 50–100 µM caused significant enrichment of the cells at S phase (Fig. 6c, d). Because its higher dose caused cell cytotoxicity, HU at 50 µM was then used for final validation.

### Enhanced crystal-cell adhesion by HU

**Fig. 7:**
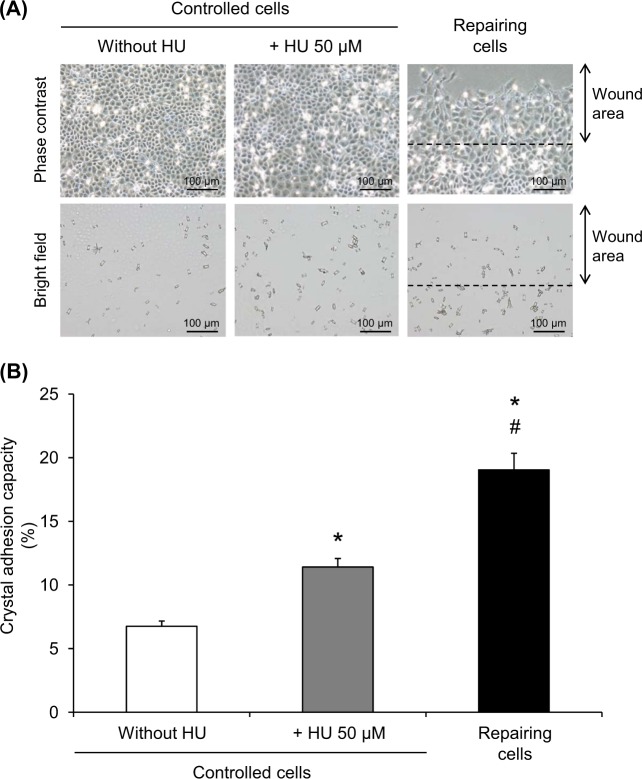
Enhanced crystal adhesion capacity of HU-treated cells. **a** COM crystal-cell adhesion assay was performed on the controlled, repairing, and HU-treated cells at 12-h post-scratch or post-treatment. Micrographs were taken by using a phase contrast microscope (original magnification = ×200 in all panels). **b** Crystal adhesion capacity of the cells was examined from at least 15 randomized high-power fields (HPFs) in each well. Each bar represents mean ± SEM of the data obtained from three independent experiments. *p < 0.05 vs. control; ^#^*p* < 0.05 vs. 50 µM HU-treated cells

To also confirm that cell cycle enrichment at the S phase contributed to the increase of crystal-cell adhesion, the cell monolayers were treated with 50 µM HU for 12 h and crystal-cell adhesion assay was performed. The remaining crystals adhered onto the cells after crystal exposure for 30 min were counted from micrographs taken (Fig. 7a) and quantitative data are shown in Fig. 7b. The results revealed that HU treatment significantly enhanced crystal adhesion capacity of renal tubular cells, although with less degree as compared to the effect observed in the repairing cells (Fig. 7).

## Discussion

Crystal adhesion capacity of renal tubular epithelial cells lining luminal side of renal tubules has been reported to facilitate nucleation of new crystals inside renal tubules, leading to crystal growth, aggregation, and finally stone formation^20–22^. Interestingly, strong evidence has been shown regarding the increased COM crystal-binding capacity of injured renal tubular epithelium that undergoes repairing process^9–13^. Nevertheless, precise mechanism(s) underlying such enhanced crystal adhesion capacity of the injured epithelial cell monolayer remained unclear. To define such mechanism(s), we first confirmed that the MDCK cell monolayer that underwent injury followed by repairing had enhanced COM crystal adhesion capacity (Fig. 1). It should be noted that the degree of such enhancement was maximal at 12-h post-scratch that was used as the optimal post-scratch time-point. The crystal adhesion capacity of the repairing cells finally returned to its basal state (as compared to the controlled cells) at 24-h post-scratch, when tissue repair was complete and the cells were expected to enter into their resting state. Subsequently, we demonstrated that the repairing cells had marked increase in cell proliferation, whereas cell death remained unchanged (Fig. 2). Regarding the optimal crystal-exposure time, 30 min provided the maximal degree of enhanced crystal adhesion capacity of the repairing cells, whereas prolonged exposure resulted to decrease in such enhancement. This was most likely due to the ability of renal tubular cells to internalize COM crystal after crystal-cell adhesion in a time-dependent manner^23–25^.

Almost all types of epithelial cells, including renal tubular cells, have ability to form cell monolayer that is capable of self-repairing after injury by inducing cell proliferation and migration to close the wound site. In addition, cell proliferation has been recognized as an important healing process in several kidney diseases affecting renal epithelial cell damage, e.g., acute kidney injury^26–29^. This process involves cell growth, DNA replication, chromosomal separation, and cell division for generating two equal daughter cells to replace lost cells and restore total number of cells after injury. In more details, cell proliferation also involves the cycle of cell division, also known as cell cycle. Following damage, the quiescent cells that had previously stopped division (G0) are stimulated to enter cell cycle at the first gap of growth (G1) to prepare for DNA replication^30–33^. Thereafter, DNA replication occurs at synthesis (S) phase and the cells proceed to the second gap of growth (G2) to proofread DNA duplication properly prior to mitosis. DNA packaging, chromosome separation, and cytokinesis subsequently arise in the mitosis (M) phase to divide the cells into two daughter halves^30–33^. After mitosis, the cells may leave this cycle for further differentiation or re-enter the new cycle for ongoing proliferating process^30–33^. Our data showed that the repairing cells had cell cycle shift from G0/G1 to S and G2/M phases (Fig. 3), indicating the enhanced proliferating activity after cell monolayer injury induced by scratch.

It was plausible that the increased crystal adhesion capacity of the repairing cells was induced by cell cycle shift. For studying cell cycle shift, modulation of cell cycle by pharmacological treatment was applied to enrich cell population into particular phase(s) of the cell cycle^34^. CsA is a cyclic undecapeptide that is widely used as an immunosuppressant following organ transplantation^35,36^. Apart from its therapeutic immunosuppression, CsA has been reported to induce cell proliferation in hepatocytes, hair epithelial cells, and gingival fibroblasts^15,37,38^. Furthermore, CsA also serves as a cell cycle modifier in various models^14,15^. Interestingly, several previous studies have demonstrated that CsA can cause cell cycle shift from G0/G1 to S and G2/M phases^14–16^, consistent to our findings observed in the repairing cells. CsA at sub-toxic doses (i.e., <10 µM)^15,39^ was thus employed to mimic cell cycle shift in the repairing cells (Figs. 3 and 4). Crystal-cell adhesion assay showed that CsA at 4 µM could enhance crystal adhesion capacity of renal tubular cells, implicating that cell cycle shift was responsible for such enhancement (Fig. 5).

In addition, HU was also used to enrich the S phase of the cells to strengthen that cell cycle shift contributed to the increased crystal adhesion capacity of the repairing cells at the injured sites. HU is a non-alkylating agent used widely for therapy of several diseases, particularly hematologic disorders^40,41^. It acts via inhibition of ribonucleotide reductase enzyme, which is involved in DNA synthesis processes. Several lines of evidence has shown that HU treatment causes cell cycle enrichment at the S phase^17–19^, consistent with our cell cycle analysis (Fig. 6). HU at a sub-toxic dose was then used for final validation. Crystal-cell adhesion assay confirmed that HU 50 µM could also enhance crystal adhesion capacity of renal tubular cells (Fig. 7).

It should be noted that although CsA and HU could enhance crystal adhesion capacity of renal tubular cells, such increase was still less than that was observed in the repairing cells (Figs. 5 and 7), indicating that there might be some other mechanisms, in addition to cell cycle shift, that could also trigger adhesion of COM crystals onto repairing renal tubular cells.

In summary, we have demonstrated herein that the repairing renal tubular epithelial cells had enhanced COM crystal adhesion capacity and underwent cell proliferation and cell cycle shift from G0/G1 to S and G2/M phases. CsA was used as a cell cycle modifier to mimic that was observed in the repairing cells. The data confirmed that CsA-treated cells, which had cell cycle shift from G0/G1 to S and G2/M phases, had increased crystal adhesion capacity similar to the repairing cells. Furthermore, HU treatment, which caused enrichment of the cells at the S phase, also enhanced crystal adhesion capacity similar to the repairing cells. These data implicate that cell cycle shift from G0/G1 to S and G2/M phases is responsible, at least in part, for increased adhesion of COM crystals on repairing renal tubular cells at the injured site.

## Materials and methods

### Cell culture

MDCK, a renal tubular epithelial cell line derived from distal tubular segment of nephron, was propagated in a growth medium containing minimum essential medium (Gibco, Grand Island, NY) supplemented with 10% fetal bovine serum (Gibco), 60 U/ml of penicillin G (Sigma; St. Louis, MO), and 60 µg/ml of streptomycin (Sigma). The cells were maintained in a humidified incubator (Thermo Fisher Scientific, Marietta, OH) at 37 °C with 5% CO_2_.

### Preparation of COM crystals

COM crystals were prepared as described previously^42,43^. Briefly, 10 mM CaCl_2_·2H_2_O in a buffer containing 10 mM Tris-HCl and 90 mM NaCl (pH 7.4) was mixed 1:1 (v/v) with 1.0 mM Na_2_C_2_O_4_ in the same buffer to make their final concentrations to 5 and 0.5 mM, respectively. The solution was incubated at 25 °C overnight. COM crystals were then harvested by a centrifugation at 2000 × *g* for 5 min. The supernatant was discarded, whereas COM crystals were washed three times with methanol. After another centrifugation at 2000 × *g* for 5 min, methanol was discarded and the crystals were air-dried overnight at 25 °C. The typical morphology of COM crystals was examined under an inverted phase contrast light microscope (Eclipse Ti-S) (Nikon, Tokyo, Japan). The crystals were decontaminated by UV light radiation for 30 min before intervention with the cells.

### Scratch assay

Scratch assay was performed according to the standard protocol^44,45^, with slight modifications. Briefly, MDCK cells were seeded in a six-well culture plate (Corning Inc., Corning, NY) at a density of 4 × 10^5^ cells/well. After confluence, the cell monolayer was scratched using a 200 µl pipette tip to generate multiple mesh-like scratches (cell-free regions) within a culture well. Subsequently, the cells were washed twice with phosphate-buffered saline (PBS) to remove debris and the cells were further incubated in the growth medium for subsequent analyses or were subjected to crystal-cell adhesion assay in a humidified incubator at 37 °C with 5% CO_2_ as detailed below. The confluent cell monolayer without scratches served as the control.

### Crystal-cell adhesion assay

To determine optimal post-scratch time-point for crystal-cell adhesion assay, the growth medium was removed at indicated time-points (2, 4, 6, 8, 12, 16, and 24 h after scratch) and replaced with the growth medium resuspended with COM crystals (100 µg crystals/ml medium). The cell monolayer was then further incubated in a humidified incubator at 37 °C with 5% CO_2_ for 60 min following the standard protocol previously established^46,47^. Thereafter, the cell monolayer was vigorously washed five times with PBS to remove the unbound crystals. The remaining crystals adhered onto the cell monolayer in the scratched well (containing repairing cells) and unscratched well (containing controlled cells) were then imaged under a phase contrast microscope (Nikon Eclipse Ti-S). These adhered crystals on repairing and controlled cell monolayers were counted from at least 15 randomized high-power fields (HPFs) per well using Tarosoft Image Frame Work software version 0.9.6 (Tarosoft; Nonthaburi, Thailand).

To determine optimal crystal-exposure time for crystal-cell adhesion assay, crystal adhesion was performed as aforementioned using 12 h as the optimal post-scratch time-point, whereas the crystal-exposure time was varied at 5, 10, 15, 20, 30, 45, and 60 min.

Degree of crystal-cell adhesion (crystal adhesion capacity) in each well was then calculated using the following formula:

Formula 1: Crystal adhesion capacity (%) = 

(number of adhered crystals in each HPF/number of cells in each HPF) × 100

### Cell death and proliferation assay

At 0- and 12-h post-scratch, the cells were detached from repairing (with multiple mesh-like scratches) and controlled (without scratches) cell monolayers in the culture well using 0.1% trypsin in 2.5 mM EDTA. The cells were resuspended in the growth medium to terminate trypsin activity and then mixed with 0.4% trypan blue solution (Gibco). Thereafter, the number of trypan blue-positive cells and total cell number were counted using a hemacytometer. Degrees of cell death and proliferation were calculated using the following formulas:

Formula 2: Cell death (%) = 

(number of trypan blue-positive cells at 12 h/total cell number at 12 h) × 100

Formula 3: Cell proliferation (%) = 

[(total cell number at 12 h−total cell number at 0 h)/

total cell number at 0 h] × 100

### Cell cycle analysis

At 12-h post-scratch, the cells were detached from repairing (with multiple mesh-like scratches) or controlled (without scratches) cell monolayers in the culture well using 0.1% trypsin in 2.5 mM EDTA and then washed twice with ice-cold PBS. The cells were then fixed with 70% ethanol on ice for at least 30 min and then washed twice with ice-cold PBS and resuspended in 100 µg/ml RNase A (Invitrogen, Paisley, UK) in PBS. After incubation on ice for 30 min, the cells were stained with propidium iodide (BD Biosciences, Franklin Lakes, NJ) at 25 °C in the dark for 5 min. Thereafter, DNA contents of the stained cells were analyzed by a flow cytometer (BD Accuri C6) (BD Biosciences)^48,49^. The histogram of cell cycle distribution was generated from 10,000 events per sample. The data were finally presented as percentages of the cells in G0/G1, S, and G2/M phases using ModFit LT 5.0 software (Verity Software House, Topsham, ME).

### CsA treatment

The confluent cell monolayers were washed twice with PBS and incubated with the growth medium containing 1, 2, or 4 µM CsA (Novartis Pharmaceuticals Corp., Basel, Switzerland) in a humidified incubator at 37 °C with 5% CO_2_ for 12 h. The cells were then subjected to cell cycle analysis and crystal-cell adhesion assay (as described with details above) comparing to the controlled cells without CsA treatment and repairing cells (from the monolayer with multiple mesh-like scratches).

### HU treatment

The confluent cell monolayers were washed twice with PBS and incubated with the growth medium containing 25, 50, or 100 µM HU (Sigma) in a humidified incubator at 37 °C with 5% CO_2_ for 12 h. The cells were then subjected to cell cycle analysis and crystal-cell adhesion assay (as described with details above) comparing to the controlled cells without HU treatment and repairing cells (from the monolayer with multiple mesh-like scratches).

### Statistical analysis

All of the aforementioned experiments were done in triplicate (three independent experiments) and the quantitative data are reported as mean ± SEM. Comparisons between two groups were done using unpaired Student’s *t*-test, whereas multiple comparisons were performed using one-way analysis of variance (ANOVA) with Tukey’s post-hoc test. *P*-value less than 0.05 was considered statistically significant.
